# Cell Lineage Infidelity in PDAC Progression and Therapy Resistance

**DOI:** 10.3389/fcell.2021.795251

**Published:** 2021-12-02

**Authors:** Antonia Malinova, Lisa Veghini, Francisco X. Real, Vincenzo Corbo

**Affiliations:** ^1^ Department of Diagnostics and Public Health, University of Verona, Verona, Italy; ^2^ Epithelial Carcinogenesis Group, Spanish National Cancer Research Centre, Madrid, Spain; ^3^ CIBERONC, Madrid, Spain; ^4^ Department de Ciències Experimentals i de la Salut, Universitat Pompeu Fabra, Barcelona, Spain; ^5^ ARC-Net Research Centre, University of Verona, Verona, Italy

**Keywords:** PDAC, pancreatic ductal adenocarcinoma, organoid culture, cell lineage, progression, therapy resistance

## Abstract

Infidelity to cell fate occurs when differentiated cells lose their original identity and either revert to a more multipotent state or transdifferentiate into a different cell type, either within the same embryonic lineage or in an entirely different one. Whilst in certain circumstances, such as in wound repair, this process is beneficial, it can be hijacked by cancer cells to drive disease initiation and progression. Cell phenotype switching has been shown to also serve as a mechanism of drug resistance in some epithelial cancers. In pancreatic ductal adenocarcinoma (PDAC), the role of lineage infidelity and phenotype switching is still unclear. Two consensus molecular subtypes of PDAC have been proposed that mainly reflect the existence of cell lineages with different degrees of fidelity to pancreatic endodermal precursors. Indeed, the classical subtype of PDAC is characterised by the expression of endodermal lineage specifying transcription factors, while the more aggressive basal-like/squamous subtype is defined by epigenetic downregulation of endodermal genes and alterations in chromatin modifiers. Here, we summarise the current knowledge of mechanisms (genetic and epigenetic) of cell fate switching in PDAC and discuss how pancreatic organoids might help increase our understanding of both cell-intrinsic and cell-extrinsic factors governing lineage infidelity during the distinct phases of PDAC evolution.

## Introduction

During embryonic development, cells progress into specialised biological units that need to perform distinct functions within their designated tissues. A cell’s “lineage” details its developmental history, which includes tightly regulated division and differentiation processes to ensure each cell meets its “fate”, i.e., differentiates into its physiologically relevant type (Furlong, 2010). Throughout this journey, cells gradually lose their potential to differentiate into alternative cell types and eventually end up in a fully differentiated state. Strict control over the processes that develop and maintain cells’ identity is crucial to ensure normal physiological functions (Lander et al., 2009). Deregulation of the programmes that maintain phenotype can lead to infidelity to cell fate and lineage conversion, with differentiated cells losing their identity and, accordingly, the expression of type/function-specific genes. Cells can either revert back to a state with increased developmental potential (de-differentiation) or switch phenotypes entirely, within or across embryonic germ layers (*trans*-differentiation) (Sancho-Martinez et al., 2012). However, cells can also *trans*-differentiate by undergoing de-differentiation first. In some situations (e.g.: response to injury), certain flexibility over the cell lineage (i.e.: plasticity) can be beneficial. For example, biliary epithelial cells (i.e., cholangiocytes) can change fate and become hepatocytes following liver damage (Deng et al., 2018). In cancer, however, the transcriptional programmes that maintain cell identity can be disrupted and eventually hijacked to drive uncontrolled proliferation (O’Brien-Ball and Biddle, 2017). Notably, suppression of cell-identity specific genes is often associated with cancer initiation and progression (Roy and Hebrok, 2015). Moreover, the ability of cancer cells to switch phenotypes can give them an evolutionary advantage that allows them to survive therapy (Yuan et al., 2019). In summary, infidelity to cell fate is an extremely important driver of cancer progression.

Pancreatic ductal adenocarcinoma (PDAC) is a lethal disease, with the lowest 5-years survival rate of all cancers (Siegel et al., 2021). Cell type infidelity seems to play an important role in PDAC initiation and progression and might even drive therapy resistance (Collisson et al., 2011; Moffitt et al., 2015; Bailey P. et al., 2016; Camolotto et al., 2018). Here, we briefly discuss the mechanisms leading to cell fate commitment within the normal exocrine pancreas (where PDAC arises from), the role of cell infidelity in cancer progression and therapy resistance and how these concepts fit within the challenging clinical context of PDAC. Finally, we focus on the role of the 3D organoid culture system and how it can contribute to elucidating the mechanisms of lineage infidelity in PDAC.

## Transcription Factors Governing Pancreas Development

To understand cell lineages in PDAC, it is important to first appreciate the cell fates in the normal and developing pancreas. The specification and maintenance of the pancreatic cell fate during embryogenesis is a highly complex and coordinated process that relies on the stepwise interplay between cell extrinsic (i.e., growth factors and morphogens) and cell intrinsic factors (i.e., transcription factors). The mature pancreas is made up of two specialised compartments: endocrine and exocrine. The endocrine compartment is composed of five types of hormone-producing cells, whose main function is to regulate nutrient homeostasis. On the other hand, the exocrine compartment is composed of acinar and ductal cells, whose main role is to produce and transport digestive enzymes, respectively.

**FIGURE 1:**
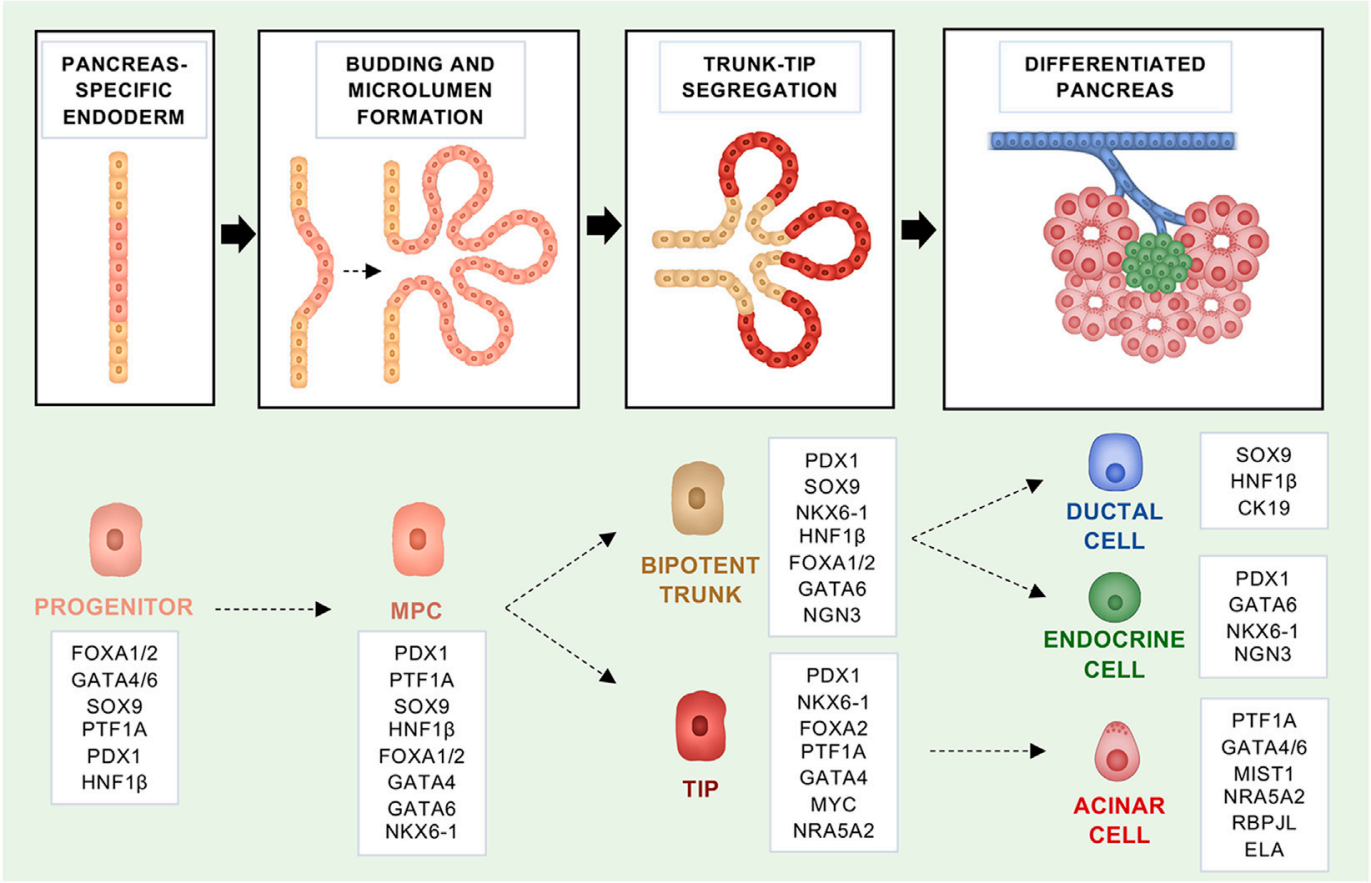
Fate regulators that govern the embryonic development of the mouse pancreas and maintain identity in the adult organ. Schematic representation of the embryonic mouse pancreas development. In boxes, the fate regulators for each developmental stage are highlighted.

Most of our knowledge of the embryonic development of the pancreas is based on mouse models owing to the ethical concerns and practical difficulties in obtaining suitable human samples, as well as to the wealth of genetic tools that can be used to study organs’ development in mice. However, the key cell fate decisions and regulators involved in the pancreas development appear to be evolutionary conserved between mice and humans (Pan and Wright, 2011). The pancreas is an endoderm-derived organ that develops from the embryonic foregut, in a region adjacent to the liver, and it is first evident in mice around embryonic day (E) 9.5 and in humans at E26 (Pan and Wright, 2011; Jennings et al., 2013; Pan and Brissova, 2014; Ghurburrun et al., 2018). In mice, pancreas development is divided into primary and secondary transitions. The primary transition takes place between E8.5 and E12.5 and includes the formation of pancreatic dorsal and ventral buds from the foregut (Figure 1). The dorsal and ventral buds contain multipotent pancreatic progenitor cells (MPCs), which can give rise to both acinar and bipotent progenitors (ductal and endocrine precursors) (Figure 1). During the primary transition, the MPCs undergo rapid proliferation and generate a stratified epithelium which, in turn, forms microlumens (Figure 1) (Villasenor et al., 2010; Pan and Wright, 2011). At E11.5, the gut tube begins to coil, bringing the two buds closer and causing them to eventually fuse and form the pancreas. During the secondary transition, between E12.5 and E15.5, the pancreatic epithelium branches and forms tip and trunk domains (Figure 1). At this stage, cell lineage allocation to the main pancreatic fates (endocrine, acinar, and ductal) begins (Zhou et al., 2007; Pan and Wright, 2011). The tip domains will end up producing acinar progenitors, whilst the trunk will produce bipotent ones (Figure 1) (Zhou et al., 2007; Pan and Wright, 2011; Villamayor et al., 2020). After E16.5, expansion of the acinar tissue is mainly driven by acinar cell replication rather than *de novo* formation of acini. Postnatally, tissue maintenance is ensured mainly by the proliferation of differentiated endocrine and exocrine cells with the replication of insulin-expressing (Dor et al., 2004; Teta et al., 2007) as well as of acinar cells (Dor et al., 2004; Hezel et al., 2006; Teta et al., 2007; Murtaugh and Keefe, 2015) gradually decreasing.

Molecularly, there are multiple extrinsic signals from the neighbouring mesoderm that control pancreas development, including fibroblast growth factors (FGFs), Wnt, retinoic acid, bone morphogenic proteins (BMPs), as well as suppression of sonic hedgehog (Shh) signalling (Hebrok et al., 1998; Martín et al., 2005; Xu et al., 2011). These signals instruct the expression of transcription factors (TFs) that confer cell fate and aid in maintaining cell identity throughout adulthood (Puri et al., 2015). The mouse genetic toolkit has helped identifying the TFs involved in the different stages of pancreatic development, including patterning of the endoderm, the specification and maintenance of the pancreatic fate, and the determination of different pancreatic cell lineages. Here, we will focus on the relevant TFs, which are causally associated with PDAC molecular subtypes, in addition to those used to generate autochthonous models of pancreatic cancer.

All pancreatic cell types derive from MPCs that are marked by the expression of *PDX1* and *PTF1A* (Figure 1) (Kawaguchi et al., 2002; Burlison et al., 2008). Of those, *PDX1* (Pancreatic and duodenal homeobox 1) is recognised as the earliest TF, expressed in the pancreas primordia (Ohlsson et al., 1993; Ahlgren et al., 1996). Nevertheless, there are TFs known to precede both PDX1 and PTF1A, and neither of the two TFs is necessary for the initial pancreatic buds’ formation (Jonsson et al., 1994; Offield et al., 1996; Stoffers et al., 1997; Krapp et al., 1998; Kawaguchi et al., 2002; Sellick et al., 2004). PDX1 can first be detected at E8.5 in mice and between E29 and E31 in humans (Sherwood et al., 2009; Jennings et al., 2013; Pan and Brissova, 2014). Even if the specification of the endoderm to a pancreatic fate does not rely on its function, *PDX1* expression is necessary for the formation of all pancreatic cell lineages and its deficiency in mouse and humans results in complete pancreatic agenesis at birth (Jonsson et al., 1994; Offield et al., 1996; Stoffers et al., 1997; Schwitzgebel et al., 2003). Later in life, high PDX1 levels are important to maintain the identity of endocrine *β*-cells and heterozygous variants of *PDX1* have been linked to the development of Mature Onset Diabetes of the Young (MODY) (Stoffers et al., 1997). In the early stages of pancreas development, the pancreas transcription factor 1a subunit PTF1A (P48) functions as part of a trimeric complex, which includes RBPJ and sustains developmental program of early pancreatic epithelium (Figure 1) (Masui et al., 2007). *Ptf1a* is first detected at E9.5 (along with *Pdx1*), and lineage tracing experiments have shown that *Ptf1a* is important for all pancreatic cell fates (Kawaguchi et al., 2002; Pan and Wright, 2011). In mice, full body *Ptf1a* deficiency results in pancreas agenesis and lethality shortly after birth (Krapp et al., 1998). Furthermore, in the absence of *Ptf1a*, cells normally contributing to the ventral pancreas are re-directed to a duodenal fate in mice (Kawaguchi et al., 2002; Burlison et al., 2008). Complementary to that, misexpression of *Ptf1a* in the early endoderm re-directs non-pancreatic endodermal cells into pancreatic precursors and determines the formation of pancreatic tissue at ectopic sites in the embryo (i.e., rostral duodenum, extrahepatic biliary system, and glandular stomach) (Willet et al., 2014). In humans, mutations in the *PTF1A* gene and an associated enhancer region have also been linked to pancreatic agenesis (Sellick et al., 2004; Weedon et al., 2014). Later during development, high *Ptf1a* expression gets restricted to the acinar progenitors and it is maintained in the differentiated acini during adulthood (Figure 1) (Puri et al., 2015). In pro-acinar cells, RBPJL replaces RBPJ in the PTF1 complex to drive the expression of the secretory digestive enzymes (Figure 1) (Hoang et al., 2016). Moreover, in the transition from MPCs to pro-acinar cells there is a critical downregulation of *c-Myc,* which has been shown to bind and repress the transcriptional activity of PTF1A (Sánchez-Arévalo Lobo et al., 2018). Other critical transcription factors for the acinar maturation are NR5A2 and MIST1 (Figure 1). NR5A2 is a nuclear receptor required during early embryonic development and active at more than one stage during pancreas development, including acinar maturation (Hale et al., 2014). *Nr5a2* deficiency results in strong reduction of endocrine cells and acini, as well as disruption in the ductal compartment (Hale et al., 2014)*.* In terms of its role in acinar cells development, NR5A2 interacts with the PTF1 complex and in its absence the remaining acinar cells do not complete differentiation (Hale et al., 2014)*.* The basic helix-loop-helix transcription factor MIST1 is required to complete acinar cell differentiation, acting downstream of PTF1A (Pin et al., 2001; Jia et al., 2008). In mice, *Mist1* deficiency results in acinar cells losing their apical-basal polarity and exocrine disorganisation (Pin et al., 2001).

While specific combinations of TFs are necessary to specify and maintain cell fates, certain TFs have a “pioneer” function: they have the unique ability to bind to closed chromatin and increase the accessibility to multiple regulatory sequences (Drouin, 2014). Members of the fork-head-box DNA-binding proteins (FOXAs) are such TFs, termed “pioneer factors”, that can bind heterochromatin and recruit additional TFs to ensure cell specification (Zaret et al., 2008). FOXA2 is expressed by the endoderm before pancreatic development (E6.5) and it is required for the development of both the liver and pancreas (Figure 1) (Lee et al., 2005; Gao et al., 2008). In the pancreas, FOXA1/2 are required to activate the pancreatic specifier *PDX1* and seem to have interchangeable roles (Gao et al., 2008).

Other relevant TFs that ensure maintenance of the pancreatic cell fate are the zinc finger TFs GATA4 and GATA6 (Figure 1). *Gata6* and *Gata4* seem to have partly redundant functions in the development of the pancreas. While full-body knockout of either *Gata4* or *Gata6* is embryonically lethal (Kuo et al., 1997; Molkentin et al., 1997; Koutsourakis et al., 1999), the pancreas-specific inactivation of either *Gata4* or *Gata6* has only mild effect on pancreas formation (Carrasco et al., 2012; Xuan et al., 2012). However, the simultaneous inactivation of both genes results in no development of the pancreas and lethality shortly after birth (Carrasco et al., 2012; Xuan et al., 2012). In mice, *Gata4/6* are expressed in the early pancreatic epithelium and throughout pancreas development (Decker et al., 2006). At late stages of pancreas development, expression of *Gata4* gets restricted to the tips of the epithelial branches and then to the acinar cells of the mature gland (Decker et al., 2006). In contrast, *Gata6* continues to be expressed by all types of pancreatic cells (Decker et al., 2006; Martinelli et al., 2013). Moreover, deletion of *Gata6* in the early pancreatic epithelium revealed the importance of the TF in maintaining acinar identity; its deletion results in restrained acinar differentiation, an increased rate of acinar cell apoptosis and acinar-to-ductal metaplasia (Martinelli et al., 2013). In humans, mutations in *GATA6* have been shown to cause pancreatic agenesis and moderate diabetes with or without exocrine insufficiency, whilst *GATA4* mutations have also been linked to neonatal and childhood-onset diabetes with or without exocrine insufficiency (Bonnefond et al., 2012; Shaw-Smith et al., 2014; Villamayor et al., 2018).

Another family of TFs that is important in pancreatic development are the hepatocyte nuclear factors (HNFs) (Figure 1). *HNF1*β is first expressed by the MPCs at E9.5 and it is required for expansion of pancreatic progenitor cells, whereas later on its expression gets restricted to ductal cells only (Figure 1) (Nammo et al., 2008; De Vas et al., 2015). *HNF1*β is critical for pancreas development and heterozygous inactivating mutations in the gene lead to MODY (Bingham and Hattersley, 2004). In mice, the homozygous deletion of *Hnf1*β in the epiblast results in pancreas agenesis owing to no formation of the ventral bud and failed expansion of progenitor cells from the dorsal bud (Haumaitre et al., 2005). Pancreas-specific inactivation of *Hnf1*β impairs expansion of MPCs by reduced proliferation and increased cell death (De Vas et al., 2015).

In contrast to acinar and endocrine cells, the regulation of the ductal fate is a little bit more elusive. This is also contributed by the heterogeneity of the pancreatic ductal system, which is composed by large ducts, small inter and intra-lobular ducts, and by intercalated ducts that insert into the acini (Flay and Gorelick, 2004; Pandiri, 2014). The use of sophisticated whole-organ 3D imaging technique applied to the adult mouse pancreas has demonstrated the heterogenous morphology of cells composing the large (cuboidal) versus smaller ducts (elongated) (Messal et al., 2019). There is some evidence that distinct developmental programmes distinguish large from intercalated ducts, however more studies are needed to elucidate concretely the lineage determinants of the ductal fate (Krapp et al., 1998; Kawaguchi et al., 2002; Hale et al., 2005; Masui et al., 2007; Nakano et al., 2015). More in general, a *Ptf1a/Nkx6-1* switch determines the tip vs trunk cell fate in MPCs (Figure 1). Whilst *Ptf1a* gets restricted to the tip compartment and determines the acinar fate, the homeobox transcription factor *Nkx6-1* becomes restricted to the trunk compartment, giving rise to the bipotent progenitors that eventually generate the endocrine and ductal cells, and it is later required for endocrine cell differentiation (Schaffer et al., 2010; Pan and Wright, 2011). Further on, endocrine and ductal progenitors are differentiated by the transient expression of *Ngn3,* which is required for the differentiation of endocrine cells. In ductal cells, the SRY-Box transcription factor, SOX9, plays a crucial role. It is expressed in mouse MPCs at E10.5 and later also by the bipotent progenitors. In adults, *Sox9* expression is maintained only by the ductal population (Figure 1) (Seymour et al., 2007). As we will see below, many of the cell fate regulators discussed so far have been used to generate conditional mouse models of PDAC. Furthermore, expression of some of those transcription factors can be used to distinguish between molecular subtypes of PDAC. In summary, mouse genetic models have allowed to precisely dissect the critical regulators of pancreatic cell type fate; most of the studies in mice have found corresponding evidence for similar roles in humans. Nevertheless, it is conceivable that some species-specific differences exist.

## PDAC and Its Cell of Origin

**FIGURE 2:**
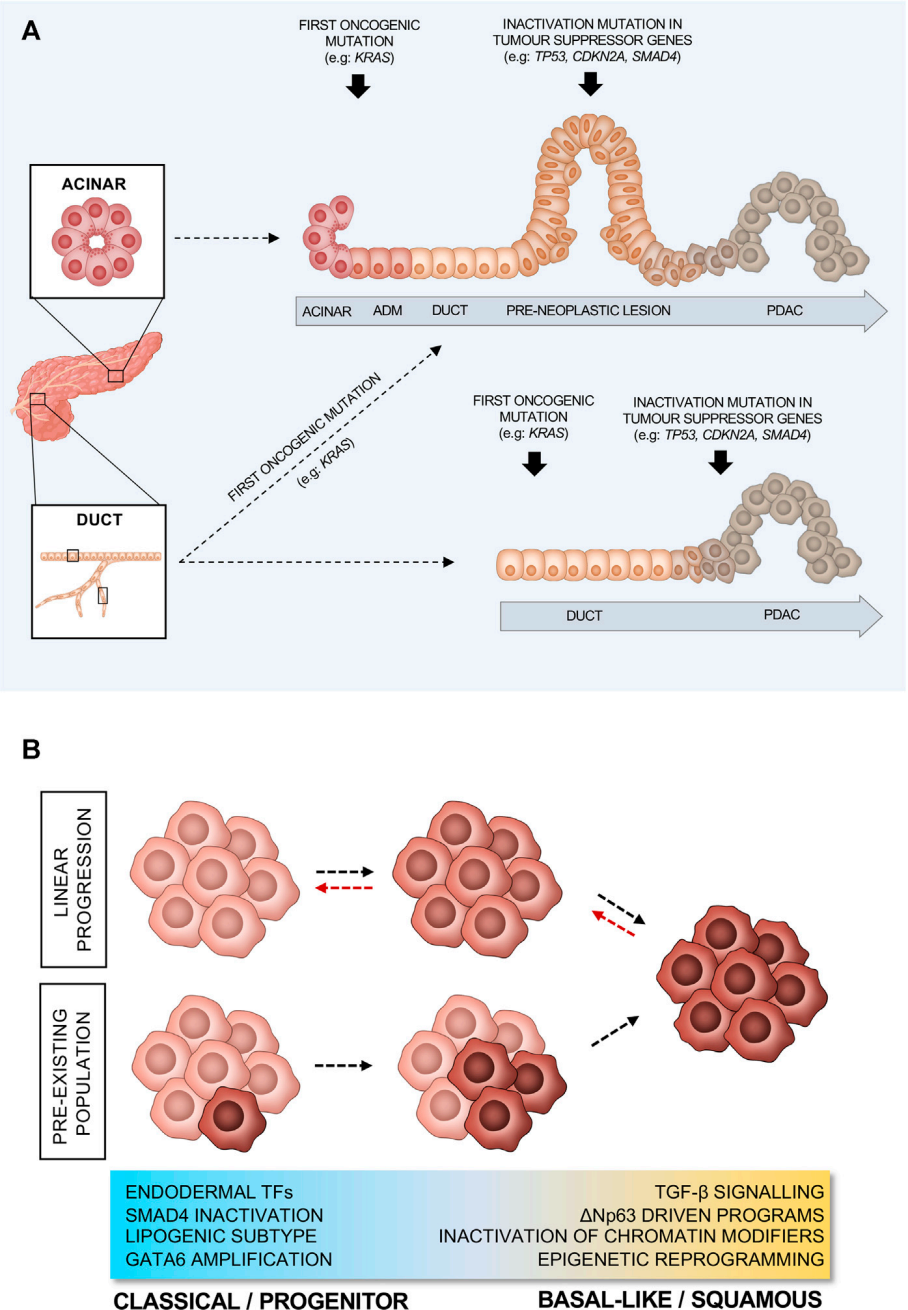
Proposed models of PDAC progression. **(A)** PDAC progression from ductal or acinar cells. **(B)** Schematic representation of different models of PDAC evolution from classical/progenitor to basal-like/squamous subtype. Red arrows indicate switch between subtypes, in response to environmental pressures.

PDAC evolves from non-invasive precursor lesions, which arise from the synergistic action of oncogenic mutations and inflammation. The majority of PDAC is believed to arise from microscopic pancreatic intraepithelial neoplasia (PanIN) (Basturk et al., 2015). However, a significant number of PDACs develop in association with large and radiographically detectable cysts that include intraductal papillary mucinous neoplasms (IPMN) and mucinous cystic neoplasms (MCN) (Basturk et al., 2015). Despite encompassing a variety of histological subtypes with specific genetic alterations, comparative sequencing of matched non-invasive neoplasms and invasive cancers has conclusively demonstrated that IPMN are a direct precursor of PDACs that are histologically indistinguishable from non-IPMN-derived tumours (Noë et al., 2020). As for other tumour entities (Chen et al., 2017; Labidi-Galy et al., 2017), early IPMN presented with remarkable heterogeneity in driver gene mutations and progression to invasive carcinoma has been associated with both loss of precancerous mutations and accumulation of further genetic abnormalities (Noë et al., 2020). In PDAC, the earliest oncogenic alteration is usually an activating mutation in *KRAS* which stimulates multiple signalling pathways to promote cell proliferation, survival, and metabolic reprogramming (Bourne et al., 1990; Suzuki et al., 2021) (Figure 2A). However, *KRAS* oncogenic activation alone is not sufficient for the development of pre-neoplastic lesions as mutations in *KRAS* can also be detected in the pancreata of people with no evidence of disease (Yan et al., 2005; Yang et al., 2017) and *Kras* oncogenic induction in adult mouse pancreas does not lead to PDAC formation (Guerra et al., 2007). Coupled with cell insult, however, *Kras* activation in mice results in the lesions that lead to PDAC (Guerra et al., 2007). Nevertheless, progression of those lesions to cancer also requires further inactivating mutations in tumour suppressor genes, such as *CDKN2A* (Maitra et al., 2003; Hezel et al., 2006) (Figure 2A). Even though these driver mutations occur in the majority of cases, PDAC tumours are characterised by extensive inter- and intra-tumour heterogeneity, which is a result of a long tail of relatively infrequent events affecting key drivers of tumorigenesis and contributing to the complex biology of this disease (Jones et al., 2008; Biankin et al., 2012; Kanda et al., 2012; Moffitt et al., 2015; Waddell et al., 2015; Bailey P. et al., 2016; The Cancer Genome Atlas Research Network, 2017; Hayashi et al., 2020).

PDAC affects the pancreatic exocrine compartment, which is made up of ductal and acinar cells. Despite the ductal morphology of the neoplastic lesions, there has been a considerable debate regarding the cell of origin of PDAC. Genetically engineered mouse models (GEMMs) have shown that PDAC can arise from pancreatic embryonic precursors, acinar cells, or ductal cells (Figure 2A). GEMMs of PDAC rely upon the pancreas-specific expression of mutant alleles and knowledge of the cell- and time-specific expression pattern of certain TFs is crucial to understanding the cell of origin. One of the most used PDAC GEMM is the KPC (*Kras* ^+/LSL−G12D^; *Trp53* ^+/LSL−R172H^; *Pdx1/p48*-Cre) model (Hingorani et al., 2003). This model is based on the Cre-Lox technology (Kim et al., 2018) that permits the conditional activation of endogenous oncogenic alleles (oncogenic activating *Kras* ^*G12D*^ mutation and a point mutation *Trp53* ^*R172H*^) in cells that express the Cre recombinase under the control of the pancreatic TFs *Pdx1* or *Ptf1a/p48* (Hingorani et al., 2003). While restricting expression of mutant *Kras* and *Trp53* to the mouse pancreatic epithelium, these models do not allow for the identification of the cell of origin as the Cre-driven recombination of the mutant alleles will happen during embryonic development (at the MPC stage), when the expression of these TFs is not restricted to a specific cell type. Conditional activation of mutant alleles in specific compartments of the adult mouse pancreas can be achieved through the use of a tamoxifen inducible Cre allele (Cre^ER^) expressed in different cell types (Pimeisl et al., 2013). This gene editing technology has made possible the generation of models, where the activation of the oncogenic alleles can be restricted either to mature acinar or ductal cells. For example, oncogenic mutations can be restricted to the ductal compartment using Cre^ER^ driven from the *Sox9* (Kopp et al., 2012; Lee et al., 2019; Flowers et al., 2021), *Hnf1ß* (von Figura et al., 2014; Bailey J. M. et al., 2016), and *Krt19* (Ferreira et al., 2017) alleles. Conversely, oncogenic insults can be restricted to mature acinar cells using *Ptf1a-* (Kopp et al., 2012; von Figura et al., 2014; Lee et al., 2019; Flowers et al., 2021), *Ela-* (De La O et al., 2008; Habbe et al., 2008; Guerra et al., 2011), or *Mist1-*driven alleles (Tuveson et al., 2006; Habbe et al., 2008; Bailey J. M. et al., 2016). Finally, next generation murine PDAC models have also been developed using a dual-recombinase system that integrates the Cre-Lox and the Flippase (Flp-FRT) recombination technologies (Schönhuber et al., 2014), which allows for sequential and independent manipulation of gene expression (Schönhuber et al., 2014; Chen et al., 2018).

When acinar cells serve as the cell of origin of PDAC, the induction of a ductal-like state is a prerequisite for transformation (Figure 2A) (Guerra et al., 2007; Habbe et al., 2008; Kopp et al., 2012; Bailey J. M. et al., 2016; Lee et al., 2019). Indeed, during this process acinar cells downregulate typical acinar markers (e.g.: *Mist1*) whilst upregulating several ductal ones (e.g.: *Sox9*). This process, termed acinar-to-ductal metaplasia (ADM) occurs upon insult (e.g., tissue inflammation), and in the presence of oncogenic *Kras,* it becomes irreversible (Figure 2A). *Kras* activation supports and maintains ADM, resulting in the preinvasive neoplasms that lead to PDAC (Liou et al., 2016). It has been shown that the ectopic expression of *Sox9* in acinar cells drives ADM (Kopp et al., 2012). Despite being quite resistant to oncogenic *Kras* induced transformation, in the presence of additional mutations, adult ductal cells have also been shown to give rise to PDAC (Figure 2A) (Bailey J. M. et al., 2016; Ferreira et al., 2017; Lee et al., 2019; Flowers et al., 2021). While histologically indistinguishable PDACs originate in mice when the same oncogenic drivers (e.g., oncogenic activation of *Kras* and/or inactivation of *Trp53* and *Fbw7*) are targeted to either acinar or ductal cells, the cell of origin seems to dictate the way the disease progresses (Bailey P. et al., 2016; Ferreira et al., 2017; Flowers et al., 2021). Acinar-derived tumours in transgenic mice exhibit a stepwise PDAC progression from PanIN lesions to frank carcinoma regardless of the type of oncogenic insult (Figure 2A) (Bailey P. et al., 2016; Ferreira et al., 2017; Flowers et al., 2021). On the contrary, oncogenic insults into adult ductal cells generate invasive PDACs without clear evidence of PanIN (Figure 2A) (Bailey P. et al., 2016; Ferreira et al., 2017; Flowers et al., 2021). However, it cannot be excluded that PanIN lesions can form when pancreatic cancer originates from ductal cells, yet they might be difficult to detect if preinvasive lesions rapidly and invariably progress to frank carcinoma (Figure 2A). Furthermore, reflecting the heterogeneity of the ductal system, mice engineered to develop tumours from adult ductal cells present with two different types of lesions growing either away from the ductal lumen (termed exophytic) or into the ducts (termed endophytic) (Messal et al., 2019). In an elegant study, Messal and others applied an innovative 3D whole organ imaging technique (termed FLASH) to the pancreata of mice, where the combination of oncogenic activation of *Kras* and the deletion of either *Trp53* or *Fbxw7* was driven in adult ductal cells by *Krt19* or *Hnf1*ß (Messal et al., 2019)*.* They showed that the morphology of the lesions did not depend upon the specific oncogenic combination, but rather on the diameter of the source epithelium, with endophytic lesions forming from ductal segments with diameter above 17 µm (Messal et al., 2019). Mechanistically, the oncogenic activation of *Kras* in ductal cells, regardless of their position in the ductal system, led to cytoskeleton changes in transformed cells with reduced apical-to-basal tension that is required for endophytic lesions to form, while the high curvature of the duct prevented inward growth in ductal segments with diameter below 17 µm. The type of lesions could be also seen in human specimens and, more importantly, both mouse and human exophytic lesions displayed a more invasive phenotype (Messal et al., 2019).

Despite being widely used to model PDAC initiation and progression, GEMMs also bear limitations, which need to be considered. For example, *Sox9* is expressed in other adult cells and ductal-derived Sox9 models have presented with other types of carcinomas (Flowers et al., 2021). Moreover, restricting *Kras* oncogenic mutation to adult *Mist1*-expressing acinar cells resulted in pancreatic tumours with mixed histological features as well as hepatocellular carcinomas (Tuveson et al., 2006). Yet, regardless of their limitations, GEMMs have shown that PDAC can arise from both acinar and ductal cells and have provided important insights into how the cell of origin affects PDAC progression (extensively reviewed in Grimont et al., 2021). However, we still do not know whether these models reflect what truly happens in patients. Reconstruction of the lineage relationships in human cancer formation requires the use of “endogenous” barcodes which can be either somatic mutations, gene variants or heteroplasmic mitochondrial DNA variants (Ju et al., 2017; Ludwig et al., 2019; Xu et al., 2019). These genetic markers can also be used in combination with single-cell sequencing technologies to trace cellular hierarchies back to the embryonic state. While still in their infancy, these methods are increasingly being used for lineage reconstruction during human organ development and have the potential of providing conclusive evidence on the cell of origin of pancreatic cancer, as well as whether PDAC predisposing mutations occur in precursor cells during embryonic development.

## Molecular Determinants of Cell Lineages in PDAC

Several studies have derived various molecular classifications of PDAC, based on bulk transcriptomic data from primary non-treated tumours as well as from cell lines (Collisson et al., 2011; Moffitt et al., 2015; Bailey P. et al., 2016; Puleo et al., 2018). What appears to be common between all classification systems is the existence of a “classical” or “progenitor” PDAC, which exhibits higher expression of pancreatic endodermal cell-fate determinants, such as *GATA6, HNF1A,* and *HNF4A* and shows slightly better prognosis (Figure 2B). On the other hand, there is a more aggressive basal-like/squamous subtype that shows loss of pancreatic identity and mostly associates with elevated expression of programmes driven by the master regulator ΔNp63, as well as with upregulation of the TGFβ signalling (Figure 2B) (Collisson et al., 2011; Moffitt et al., 2015; Bailey P. et al., 2016; Puleo et al., 2018). Genetic and non-genetic dysregulations of gene expression programmes involved in the maintenance of pancreatic cell identity are integral drivers of PDAC molecular subtypes. In their seminal manuscript, Bailey and others (Bailey P. et al., 2016) showed the association between reduced expression (through gene hypermethylation) of the endodermal cell fate determinants (*PDX1, GATA6*, and *HNF1*β) and the basal-like phenotype. In particular, GATA6 has been demonstrated as a critical regulator of the classical programme and thus a valid surrogate biomarker of the classical subtype (Martinelli et al., 2017; Aung et al., 2018). However, it has been recently shown that, while necessary, GATA6 loss is not sufficient to drive the basal phenotype (Kloesch et al., 2021). Further downregulation of other endodermal fate determinants such *HNF1A* and *HNF4A* is also needed for the complete switch from classical to squamous/basal-like subtype (Kloesch et al., 2021). This is supported further by the fact that HNF4A loss also causes a switch to a squamous metabolic profile in human PDAC cell lines (Brunton et al., 2020). Epigenetic reprogramming, due to alterations in epigenetic modifiers might also favour gradual loss of the endodermal cell fate. This is supported by the fact that basal-like/squamous tumours exhibit alterations in epigenetic modifiers and transcription master regulators, such as *ARID1A* and *MYC* (Figure 2B)*.* In a recent multiregional sampling analysis of primary and metastatic PDACs, the integration of histology, expression profiling, and DNA sequencing revealed the enrichment of clonal mutations in chromatin modifiers (e.g., *ARID1A*, *KMT2C*, *KMT2D*, and *KDM6A*) in tumours with basal-like/squamous features (Hayashi et al., 2020). Furthermore, aberrant activation of *MYC,* due to gene amplification, drives PDAC progression by activating cell proliferation, survival programmes (Dang, 1999; Pelengaris et al., 2002), and metabolic reprogramming (Dey et al., 2020). Accordingly, the frequency of *MYC* amplification is higher in advanced stage PDACs and in tumours with basal-like features (Hayashi et al., 2020). However, the effect of *MYC* amplification on cellular lineage seems to be context-dependent as induced overexpression of *MYC* in PDAC cells conferred the basal-like/squamous phenotype exclusively in the background of chromatin modifier genes inactivation (Hayashi et al., 2020). The SWI/SNF subunit AT-rich interactive domain ARID1A regulates the expression of *Sox9* to maintain the ductal fate while its loss drives aggressive PDACs (Kimura et al., 2018). The loss of another epigenetic regulator, the X-chromosome encoded histone demethylase KDM6A, activates gene networks regulated by p63 and MYC that promote squamous-like and poorly differentiated PDAC with sarcomatoid features (Andricovich et al., 2018). Interestingly, Andricovich and others (Andricovich et al., 2018) demonstrated that gene expression changes resulting from the loss of *Kdm6a* are independent from the enzyme’s demethylase activity but are rather due to changes in the activity of super-enhancers. Similarly to the loss of *Kdm6a*, the pancreas-specific deletion of *Hnf1a* synergises with mutated *Kras* to induce PDAC lesions with sarcomatoid features as well as a molecular phenotype that aligns with human basal-like/squamous tumours (Kalisz et al., 2020). Mechanistically, HNF1A recruits KDM6A at functional genomic sites in acinar cells to activate differentiation and suppress oncogenic pathways (Kalisz et al., 2020). More evidence for the epigenetic reprogramming of PDAC has been provided by Somerville et al. (Somerville T. D. D. et al., 2020), showing aberrant expression of the transcription factor zinc finger protein (ZBED2), which seems to downregulate the pancreatic progenitor cell fate. In addition to the expression of transcription factors and the genetic inactivation of chromatin modifiers, distinct methylation patterns of repetitive elements can be used to distinguish classical from basal-like PDAC (Espinet et al., 2021). Tumours showing low levels of DNA methylation at these elements (defined as MC2, methylation cluster 2) display increased Interferon-response signatures, a pro-inflammatory microenvironment, and associate with the basal-like phenotype (Espinet et al., 2021). Moreover, through oxidative bisulfite sequencing of archival samples, Eyres et al. (2021) have recently found that the basal/squamous-like phenotype is a direct result of epigenetic silencing of regulator of the classical programme. In basal-like/squamous tumours, the authors found that TET2-maintained levels of 5-hydroxymethylcytosine (5 hmc) are significantly reduced at genetic loci which promote the classical gene programme (such as *GATA6*). This further supports the classical programme as the “default lineage” that is epigenetically silenced to drive a phenotype switch towards the basal-like/squamous cell lineage.

While the dichotomisation into two subtypes has the perceived advantage of simplifying biomarker and functional studies, there is increasing evidence that cells with classical and basal-like features co-exist in the same tumour (Figure 2B) (Puleo et al., 2018; Porter et al., 2019; Chan-Seng-Yue et al., 2020; Hwang et al., 2020; Juiz et al., 2020; Nicolle et al., 2020). This evidence has been generated from analyses of both human tissues (Puleo et al., 2018; Porter et al., 2019; Chan-Seng-Yue et al., 2020; Hwang et al., 2020; Raghavan et al., 2021) and *ex vivo* cultures (Porter et al., 2019; Juiz et al., 2020; Nicolle et al., 2020; Raghavan et al., 2021) and implies that molecular classification systems should account for this phenotypic heterogeneity for a better prediction of patient outcomes. Accordingly, Nicolle and others have recently shown the benefit of classifying patients based on a continuum of phenotypes rather than on two non-overlapping subtypes (Nicolle et al., 2020). Despite the observation of co-existence of subtypes within the same tumour, the question remains as to whether those are two interconverting cell types, different entities, or bear a precursor-to-product relationship. There is some evidence to suggest that PDAC progression is associated with accumulation of basal-like cells (Figure 2B). Enrichment of basal-like/squamous cells has been observed in advanced stages of the disease (Chan-Seng-Yue et al., 2020) as well as in post-treatment tumours (Hwang et al., 2020). This is also supported by *in vitro* data showing enrichment of basal state post-treatment with FOLFIRINOX (Porter et al., 2019) and that PDAC cell lines exist on a continuum, suggesting linear evolution from classical to basal-like/squamous PDAC. However, there is also evidence to suggest that the cell of origin affects PDAC’s progression. Support to this hypothesis is given by a recent study proposing that, in mice, the cell of origin can also influence subtypes as ductal cell-derived PDAC are enriched for basal-like signatures, whilst the acinar derived ones are enriched for classical gene signatures (Flowers et al., 2021). In keeping with this observation, the MC2 methylation subtype described by Espinet and others (Espinet et al., 2021) and aligning with the transcriptomic basal-like subtype is suggested to derive from ductal cells. Furthermore, a recent manuscript demonstrating the presence of a rare ΔNp63^+^ ductal cell population in the normal human pancreas raises the possibility that these might represent a cell of origin for tumours with basal-like/squamous features (Martens et al., 2021). Moreover, cell reprogramming, as a driver of basal-like/squamous PDAC, might have a cell-dependent context. For example, in ductal cells only, loss of *ARID1A* appears to promote *MYC* driven gene programmes and the formation of cystic PDAC (Wang et al., 2019).

In summary, basal-like cells appear to accumulate in PDAC as the tumour progresses or under the selective pressure of certain chemotherapeutics (Figure 2B). Cells with basal-like features might originate from classical cells via genetic and non-genetic dysregulation of pancreatic transcriptional programmes. Alternatively, classical, and basal-like cells in PDAC might have different ontogeny. Finally, we cannot exclude that in some instances they represent interconverting cell types depending on microenvironmental conditions (Figure 2B). None of these hypotheses is necessarily mutually exclusive of the others.

Single cell and spatial transcriptomics promise to provide further insights into the evolution of PDAC. Recent studies have used single-cell RNA sequencing of either biopsies or organoids coupled with multiplex immunofluorescence to reveal that classical and basal programmes co-exist even at the cellular level (Juiz et al., 2020; Raghavan et al., 2021). Finally, these techniques might help elucidate better the role of the microenvironment in influencing PDAC subtypes.

## The Influence of the Tumour Microenvironment on PDAC Subtypes

PDAC is characterised by a prominent stromal component, which can make up to 80% of the tumour mass (Erkan et al., 2012). *In silico* micro-dissection of transcriptomic data from bulk PDAC tissues by Moffitt and others (Moffitt et al., 2015) identified two major stromal subtypes, namely the “normal” and “activated” subtypes, with the latter enriched for expression of inflammatory cytokines and preferentially associated with the basal-like subtype. Furthermore, single-cell RNA sequencing has also revealed that the tumour microenvironment (TME) appears to be just as heterogenous as the tumour cells themselves, and that it also seems to influence subtypes (Peng et al., 2019; Raghavan et al., 2021).

The TME consists predominantly of cancer-associated fibroblasts (CAFs), but there is also an abundance of immune and endothelial cells (Murakami et al., 2019). CAFs are largely responsible for the desmoplastic reaction in PDAC, as they secrete multiple extracellular matrix (ECM) components (Tian et al., 2019; Sperb et al., 2020). They mainly arise from quiescent pancreatic stellate cells (PSCs) that become activated in response to injury, from tissue-resident fibroblasts, and from mesenchymal stromal cells recruited to the tumour site (Öhlund et al., 2014; Sperb et al., 2020; Gorchs and Kaipe, 2021). CAFs have been invariably associated with pro-tumorigenic functions, and the dense desmoplasia they produce was historically considered as both a physical and a biochemical barrier to the delivery of therapies to tumour cells (Olive et al., 2009; Erkan et al., 2012). However, CAF depletion in experimental mouse models surprisingly led to worse prognosis and higher tumour aggressiveness (Rhim et al., 2014; Özdemir et al., 2014). In mice, depletion of CAFs at different stages of PDAC evolution invariably led to acceleration of the disease, poor differentiation of epithelial cells, and reduced animals’ survival (Rhim et al., 2014; Özdemir et al., 2014). In this context, there was substantial remodelling of other relevant microenvironmental features. Indeed, Özdemir et al. showed that genetic depletion of αSMA positive cells increased survival of experimental mice upon blocking of the immune checkpoint receptor CTLA-4 (Özdemir et al., 2014). Blockade of the sonic hedgehog axis, either pharmacologically or genetically (Rhim et al., 2014), led to tumours with increased vasculature and, accordingly, superior sensitivity to vascular endothelial growth factor (VEGF) inhibition. Finally, myofibroblast-specific deletion of type 1 collagen in a mouse model of PDAC accelerated progression of the disease resulting in more undifferentiated tumours’ histology (Chen et al., 2021). These preclinical findings might explain the failure of clinical trials testing the use of sonic hedgehog inhibitors (stroma depleting agents) in association with chemotherapy, which resulted in progression of disease and poorly differentiated tumours (Kim et al., 2014; Ko et al., 2016). Furthermore, they suggest that targeting of certain stromal elements might lead to increased sensitivity of PDAC to therapeutic agents (e.g., immune checkpoint inhibitors) that otherwise have no effects. Overall, these data suggest that the influence of CAFs on the tumour behaviour is much more complex than initially anticipated. In terms of lineage plasticity, CAFs might participate in the process by secretion of growth factors, cytokines, ECM components and other signalling molecules (Hass et al., 2020). For example, CAFs represent a prominent source of TGFβ1, which appears to drive PDAC cells to a more proliferative and undifferentiated phenotype, consistent with the role of TGFβ signalling in the basal-like/squamous subtype (Ligorio et al., 2019). Recent studies on mouse and human PDACs have revealed different CAFs subpopulations with distinct functions (Öhlund et al., 2017; Elyada et al., 2019). Most notably, they found that the inflammatory CAFs (iCAFs), characterised by high expression of inflammatory interleukins, act to promote tumour progression and are located distally from the neoplastic glands (Öhlund et al., 2017; Biffi et al., 2019). Myofibroblast CAFs (myCAF), which are in the vicinity of neoplastic cells, are instead characterised by high expression of αSMA and appear to restrain tumour growth (Öhlund et al., 2017; Biffi et al., 2019; Bhattacharjee et al., 2021). A recent study demonstrated both anti- and pro-tumorigenic function for myCAFs in the context of metastatic PDAC (Bhattacharjee et al., 2021). The pro-tumorigenic effects of myCAFs result from their production of hyaluronan, which promotes cancer proliferation, whilst the type 1 collagen produced by myCAFs acts to suppress the tumour, which is in line with findings from Chen and others (Bhattacharjee et al., 2021; Chen et al., 2021). Moreover, an antigen-presenting CAF subpopulation, characterised by its ability to activate CD4^+^ T cells has also been found (Elyada et al., 2019). Finally, the secretome of basal-like/squamous PDAC cells can polarise PSCs and fibroblasts towards the iCAF phenotype (Somerville T. D. et al., 2020), but how distinct CAF populations affect tumour subtype is still unclear.

Immune cells, and tumour associated macrophages (TAMs) in particular, are another important component of the PDAC TME. Macrophages are recruited to the tumour via signalling from the cancer cells, where they become TAMs (Yang et al., 2021). However, just like CAFs, resident macrophages can also become TAMs (Yang et al., 2021). TAMs participate in establishing a high immunosuppressive environment and their density within the PDAC TME is correlated with worse prognosis (Habtezion et al., 2016; Hu et al., 2016). In several preclinical studies, TAM depletion has been shown to reduce metastatic burden, improve response to the chemotherapy drug gemcitabine (Buchholz et al., 2020), and alter gene programmes that define the basal-like/squamous subtype (Candido et al., 2018). Using single cell RNA sequencing of patient metastases, Raghavan et al. (2021) have classified TAMs in three different subtypes: monocyte-like, phagocytic, and angiogenesis-associated TAMs. The authors also found an association between basal-like tumours and phagocytic TAMs, and between classical and angiogenesis-associated TAMs, suggesting reciprocal influences between epithelial and stromal subtypes.

Tumour associated neutrophils (TANs) are the other important component of the PDAC immune microenvironment (Jin L. et al., 2021). Neutrophils are recruited by the tumour via chemokines, most notably CXCs (Hosoi et al., 2009; Steele et al., 2016; Nywening et al., 2018). Just like TAMs, they also support the proliferation of neoplastic cells, promote an immunosuppressive environment and facilitate distant metastases (Stromnes et al., 2014; Lianyuan et al., 2020). Depletion of neutrophils in a KPC mouse model reduces metastatic burden and causes a switch from squamous to progenitor subtype (Steele et al., 2016). Furthermore, preventing neutrophil recruitment in a PDAC mouse model led to recruitment of T-cells and tumour-suppression (Chao et al., 2016). Pharmacological suppression of neutrophils also makes mouse PDAC more vulnerable to immune checkpoint blockade (Nielsen et al., 2021). In wound healing and *trans*-well assays, neutrophils from PDAC patients promote tumour cell migration and invasion, whilst neutrophils from healthy individuals cannot (Jin W. et al., 2021). Moreover, it seems that the TME of basal-like/squamous tumours is characterised by an increased infiltration of neutrophils that is at least partially driven by secretion of Cxcl1 by squamous-instructed iCAFs (Somerville T. D. et al., 2020). All these studies show that neutrophils are pro-tumorigenic, yet how they impact on subtypes and lineages in PDAC is unclear.

Finally, endothelial cells are also found in the PDAC TME (Feig et al., 2012). Generally, PDAC is an extremely hypoxic tumour and is poorly vascularised (Zhang et al., 2018). Under hypoxia, hypoxia-inducible factor (HIF-1α) drives VEGF upregulation which promotes tumour angiogenesis, proliferation and metastases (Zhang et al., 2018). Yet, vascular remodelling in PDAC has also been shown to improve delivery of therapies and activation of T cells (Ruscetti et al., 2020). However, the role of endothelial cells and how they support tumour progression and subtypes is still unclear.

## Lineage Infidelity and Therapy Resistance

The capability of cancer cells to move across cell states (i.e., fate plasticity) is a source of cell heterogeneity that cancers employ to survive drug treatment (Quintanal-Villalonga et al., 2020). Infidelity to the cell lineage, in particular, has been implicated in therapeutic resistance in multiple solid cancers (Seldin and Macara, 2020). Most prominently, therapy resistance in prostate adenocarcinoma can be driven by *trans*-differentiation of cancer cells into a neuro-endocrine phenotype (Hu et al., 2015). Similarly, EGFR-positive non-small cell lung tumours acquire resistance to anti-EGFR therapies also by *trans*-differentiation into a neuroendocrine phenotype (Oser et al., 2015). In PDAC, the contribution of lineage infidelity to therapy resistance is less clear. This is due to the difficulties in procurement of tissues from patients undergoing treatment. Thus, most of our knowledge related to mechanisms of escape to treatments relies on preclinical works. Recently, single nucleus RNA sequencing analysis of archival samples from post-treatment tumours has revealed an enrichment of basal-like cells in the post-treatment setting, consistent with the more aggressive nature of basal-like/squamous tumours (Hwang et al., 2020). Furthermore, Hwang and others (Hwang et al., 2020) also reported an enrichment of neuroendocrine transcriptional programmes in post-treatment tumours, suggesting that neuroendocrine *trans*-differentiation might play a role also in this cancer type. Given their profound differences, it is not unexpected that the two main cell lineages of PDAC display different pharmacological sensitivity. In his seminal work, Collisson reported that classical cell lines were more sensitive to the epidermal growth factor receptor (EGFR) inhibitor erlotinib, whilst basal-like (defined as quasi-mesenchymal) cells exhibited sensitivity to gemcitabine (Collisson et al., 2011). The squamous/basal-like tumours exhibit a glycolytic metabolic profile (as opposed to the lipogenic profile of the classical cells) (Bailey P. et al., 2016), which is susceptible to glycogen synthase kinase 3β (GSK3β) inhibition (Brunton et al., 2020). In this study, inhibition of GSK3β eventually resulted in resistance; however, the resistant cell lines were also susceptible to porcupine inhibition (inhibition of WNT ligand production) (Brunton et al., 2020). In addition to preclinical studies, differential sensitivity of PDAC subtypes to available chemotherapeutic regimens has been also demonstrated, with classical tumours reported to be more sensitive to FOLFIRINOX and, in contrast with the findings from Collisson et al., to gemcitabine (Martinelli et al., 2017; Aung et al., 2018; Nicolle et al., 2021). Recently, a transcriptomic signature of elevated replication stress generated from analysis of patients-derived cell lines was found enriched in basal-like/squamous tumours and predicted responses to cell cycle checkpoint inhibitors in cell lines and organoids (Dreyer et al., 2021). These findings will be tested by the ongoing clinical trial PRIMUS004 (ISRCTN16004234).

Even if there is a consensus that the basal-like/squamous subtype is a more aggressive form of the disease in the setting of an early stage and resectable disease (Bailey P. et al., 2016), it should be noted that the classical PDAC subtype is as lethal as the basal-like. Furthermore, this dichotomisation is not informative of patients prognosis in a more advanced setting (Chan-Seng-Yue et al., 2020), where more complex and hybrid cell states also seem to emerge (Hwang et al., 2020). Therefore, it is more likely the ability of cells to switch between lineages and subtypes provides them with a superior advantage to escape from different types of selective pressures (Figure 2B). Given the profound biological differences between subtypes and their unique therapeutic vulnerabilities, it is conceivable that a viable strategy to achieve deeper and durable responses in PDAC might be the identification of targets that prevent subtype switching.

While basal-like and classical subtypes in PDAC likely reflect two different epithelial differentiation programs, the epithelial-mesenchymal transition (EMT) refers to a process whereby epithelial cells acquire mesenchymal traits (Kalluri and Neilson, 2003) that favour cell dissemination and metastatization (Wang et al., 2017). The contribution of EMT to therapeutic resistance in PDAC has been investigated more in depth than lineage infidelity. As for other solid malignancies, upregulation of the EMT program in PDAC has been shown to be associated with poor prognosis and therapy resistance (Javle et al., 2007; Arumugam et al., 2009; Weadick et al., 2021). EMT is activated by TGFß signalling, which in turn tends to be upregulated in more aggressive basal-like/squamous PDACs. EMT has also been shown to be regulated by the classical transcription factor GATA6, which directly represses EMT genes while positively regulating pro-epithelial genes (Martinelli et al., 2017). Accordingly, single-cell analysis of human PDAC cells has demonstrated that basal-like and classical programs are positively and negatively associated with EMT, respectively (Chan-Seng-Yue et al., 2020). Whether the switch from classical to basal-like phenotypes precedes the acquisition of a full EMT phenotype by PDAC cells needs to be clarified. EMT might play an important role in therapy resistance as it is associated with gemcitabine resistance both in cell lines and patients (Weadick et al., 2021). This is supported by data in KPC mice showing that EMT inhibition via knockout of the EMT-inducing TFs *Twist1* and *Snai1* improved response to gemcitabine and increased survival (Zheng et al., 2015).

## Organoids: A 3D Platform to Model PDAC Initiation and Progression

**FIGURE 3:**
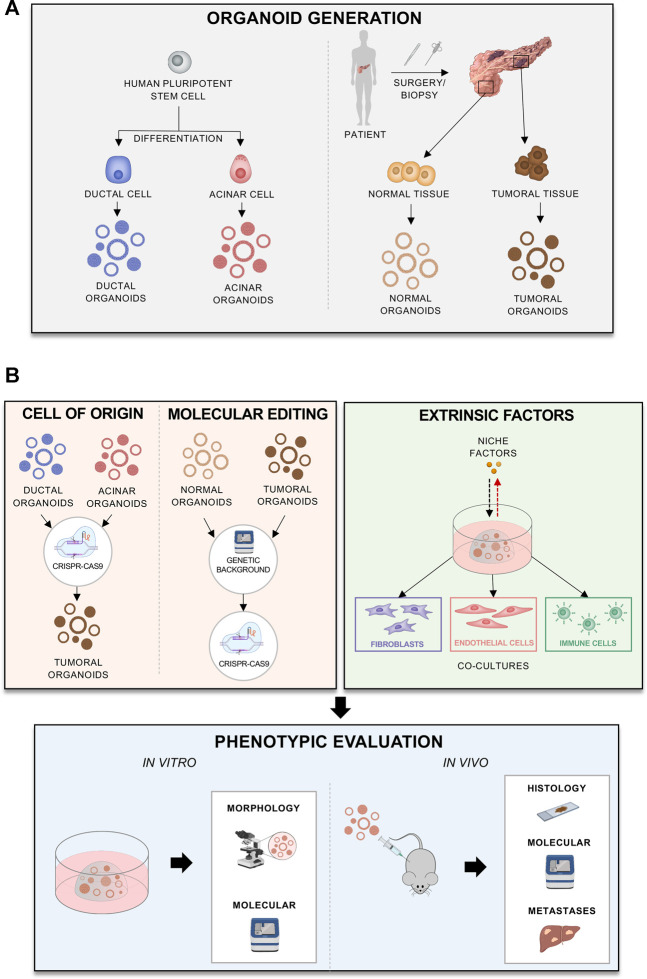
Different applications of organoids to model progression **(A)** Different methods and sources for organoid derivation. **(B)** Generation of different organoid models, reflecting cell of origin, mutations of interest, patients’ background, or effects of extrinsic factors. The models can be phenotypically evaluated *in vitro* or *in vivo*.

While it seems clear that a loss of endodermal commitment is a feature of aggressive PDAC phenotypes, the mechanisms leading to this lineage infidelity are still elusive. We believe that the pancreatic organoids culture system offers a unique opportunity to model the contributions of the cell of origin as well as of tumour intrinsic and extrinsic factors to the definition of PDAC cell fate. Organoids are a 3D culture system, where epithelial cells can be cultured in a semi-solid medium supplemented with growth factors and morphogens that collectively recreate the *in vivo* stromal *niche* (Drost and Clevers, 2018; Seino et al., 2018). Organoids can be derived directly from adult primary cells, either from healthy or diseased pancreata, and from human pluripotent stem cells (iPSCs) permitting expansion of the epithelial compartment even from limited amount of material (Figure 3A). In the field of epithelial tumours, this culture system has recently become the alternative to 2D cell lines, as organoids have been shown to preserve better the histological and genetic features of the parental tumours (Weeber et al., 2015; Baker et al., 2016). Organoids can have clinical implications as they mimic patient response and can be used to identify patients that would benefit from certain treatments (Tiriac et al., 2018). Additionally, organoids derived from embryonic pancreatic cells can also contribute to understanding better the human pancreatic development and lineage relationships, which are altered in PDAC (Balak et al., 2019). Finally, organoid cultures can be established and propagated (albeit for a limited time) from adult pancreatic exocrine cells, which allows evaluating the contribution of individual genes and their influence on the PDAC tumorigenic process by the stepwise introduction of genetic alterations through genome editing approaches (Figure 3B) (Greggio et al., 2013; Huch et al., 2013; Huch and Koo, 2015; Baker et al., 2016). Indeed, the Sato group (Seino et al., 2018) has demonstrated the feasibility of genetically engineering human normal pancreas organoids through the sequential introduction of the typical PDAC alterations (*KRAS, TP53, CDKN2A, SMAD4*) and that only quadruple mutant organoids generated lesions histologically resembling human PDAC when transplanted in immunodeficient mice (Seino et al., 2018).

With regards to the cellular origin of PDAC, organoids might provide a human alternative to GEMMs (Figure 3B). Despite the similarities between humans and mice, there are differences between the two species in pancreas anatomy and development. Furthermore, murine tumours tend to display less genetic diversity and complexity than human cancers. To elucidate the role of the cell origin in the disease progression, human acinar and ductal organoids can be derived from iPSCs and transformed to model PDACs derived from each cell type (Figure 3A) (Breunig et al., 2021; Huang et al., 2021). This has shown that different oncogenic mutations, such as *KRAS* and *GNAS*, affect tumorigenesis differently depending on the cellular context (Huang et al., 2021). *In vivo* transplantation of acinar organoids with *KRAS* mutations caused cancer lesions more effectively, whilst *GNAS* mutations in ductal ones caused cystic outgrowths (Figure 3B). The study from Huang and others (Huang et al., 2021) also shed some light on how initiating cancer mutations affect cell identity in PDAC. In acinar organoids, *KRAS* mutations caused silencing of acinar-specific genes (*PTF1A*) and upregulation of the ductal *SOX9*. In ductal organoids, however, *KRAS* upregulated *SOX9, NKX6-1* and *PDX1*, suggesting re-direction of cells towards a progenitor state (Huang et al., 2021). In another elegant study, the Kleger’s laboratory developed a two-phase protocol to differentiate human PSCs into pancreatic ductal-like organoids (PDLO) which recapitulated features of mature ductal cells (Breunig et al., 2021). PDLOs were then used to explore the cell-context specific effects of oncogenic drivers (either alone or in combination) on the development of dysplastic and cancerous lesions (Breunig et al., 2021). Upon orthotopic engraftments of PDLOs engineered to carry different combinations of oncogenic insults, they found that PDLOs carrying *KRAS* activating mutation generated heterogeneous dysplastic lesions, while PDLOs with simultaneous activation of *KRAS* and loss of *CDKN2A* generated de-differentiated tumours (Breunig et al., 2021). When PSCs were engineered to express the oncogenic *GNAS* ^*R201H*^ variants, PDLOs formed large cysts *in vitro* and IPMN-like structure upon engraftment (Breunig et al., 2021). This is in line with the prevalence of GNAS mutations in IPMN (Furukawa et al., 2011; Hosoda et al., 2015) and the observations from Huang et al. (Huang et al., 2021). The same *in vitro* and *in vivo* cystic phenotype was observed when PDLOs were established from iPSCs of a patient suffering from McCune-Albright syndrome (MAS), which is caused by postzygotic mosaic *GNAS* mutations (Breunig et al., 2021). This work exemplifies the possibility of using organoids to assess the impact of individual patients’ genetic background on inception and progression of the disease and strengthen the evidence that a complex interplay between the oncogenic mutations and cellular context dictate the way disease progresses (Figure 3B).

Organoids established from tumour resections also provide a powerful platform to study cell autonomous processes that affect lineage commitment and malignant behaviour (Figure 3A). For example, organoids have been used to show how *MYC* copy number gain drives PDAC progression. *MYC* amplification is associated with poor prognosis and advanced disease. To model how MYC affects PDAC, Hayashi et al. (2020) overexpressed *MYC* in patient derived organoids with and without deleterious mutations in chromatin modifier genes, such as *ARID1A*. The authors showed that *MYC* overexpression induces squamous features, only when combined with mutations in the chromatin modifier genes. Moreover, organoids can be genetically modified to study effects of different mutations (Figure 3B): for examples Seino et al. (2018) introduced different driver mutations into organoids in order to examine whether cancer driver mutations, alone, can confer niche factor dependency. Thus, organoids provide a personalised platform to study how an individual’s genetic background affects functional perturbations, which also include therapeutic treatment.

The TME has a dramatic influence on PDAC progression, and therefore it is important to understand how stromal signals affect tumour cells and vice versa. *In vivo* experiments of organoid transplantation in mice have demonstrated that the local microenvironment drives PDAC subtypes (Miyabayashi et al., 2020). As the PDAC stroma supports the tumour by producing ligands, elucidating appropriate ligand-receptor relationships between the stroma and the tumour might provide novel drug targets. This has been exemplified by a recent study on single cell RNA-Seq data from primary and metastatic tumours, identifying multiple potential ligand-receptor relationships and opening avenues for targeted therapies (Lee et al., 2021).

Organoids can provide a powerful platform to study the interactions between the stroma and tumour cells either through modification of the culture medium or by coculturing neoplastic cells with the different microenvironmental components (Figure 3B). The TME can be partially recreated *ex vivo* using organoid-based coculture systems (Figure 3B). As an example, Öhlund and others demonstrated that the co-cultivation of mouse tumour organoids with PSCs trigger the deposition of stroma *ex vivo* (Öhlund et al., 2017). Moreover, this co-culture system permits modelling of the different CAF subtypes, which show different functions and influence on tumour behaviour (Öhlund et al., 2017; Biffi et al., 2019). Using organoid-based co-cultures, Feldmann et al. (2021) have shown that CAFs expressing the transcription factor *Prrx1* can induce PDAC cells transition towards a mesenchymal phenotype. Moreover, there appears to be a stromal niche dependency of PDAC organoids that also associates with expression of endodermal transcription factors. Seino et al. (2018) revealed three subtypes of PDAC organoids, with distinct dependency on Wnt ligands. PDAC organoids classified as “classical” were reported to be more dependent for their propagation on the supplementation of Wnt ligands, either exogenously or through cocultivation with CAFs (Seino et al., 2018). Interestingly, suppression of *GATA6* expression rendered organoids less reliant on exogenous Wnt supplementation. Thus, it is likely that stromal niche factors play a role in maintaining the endodermal commitment and that depletion of certain signalling cues from the organoid-rich media would allow for modelling progression associated with depletion of stromal elements (Figure 3B).

Organoid co-culture systems can also provide a platform to elucidate the role of the other components of the stroma on the tumour subtype, including immune cells (Figure 3B). Co-culture of PDAC organoids with autologous lymphocytes and CAFs showed activation of a myCAFs phenotype and infiltration of the lymphocytes towards the tumour cells (Tsai et al., 2018). Thus, the co-culture system can incorporate multiple cell types and help modelling the interactions between the human TME and cancer cells (Figure 3B).

While there is a considerable excitement about the possibility that tumour organoids facilitate translational research and even become part of the clinical decision-making process, they are models and therefore, are imperfect. The limitations of the culture system, which restrain its wider adoption by the scientific community and the implementation in clinical practice, should be acknowledged. One important bottleneck of the technology is the success rate in the derivation of cultures. While the establishment of organoid cultures is undoubtedly more efficient than 2D culture methods, the methodology still needs optimisation to enable the systematic and timely derivation of *ex vivo* cultures to meet clinical criteria. Furthermore, we do not know whether failure in generating cultures is driven by specific genotypes/phenotypes that cannot be captured efficiently or rather due to characteristics of the specimens, such as the neoplastic cell content. The optimisation of the culture conditions seems necessary also to limit across-laboratory variability due to the use of the animal-derived matrices that suffer from batch-to-batch variability and undefined composition. Beyond the standardisation of the ECM components, considerable attention has been recently given to the growth medium composition due to the presence of elevated concentrations of growth factors and pathways inhibitors, which are potential confounders of functional perturbation experiments and substantially contribute to the elevated costs of the technology. Moreover, single cell sequencing has shown that organoids can drift away molecularly from their original tissue by becoming more “classical”, even when derived from basal-like/squamous tumours (Raghavan et al., 2021). Given the differential responses to available chemotherapy regimens reported for the two molecular subtypes in the adjuvant setting (Collisson et al., 2011; Aung et al., 2018; Porter et al., 2019; Brunton et al., 2020; Nicolle et al., 2021), the inability of organoids to faithfully replicate patients’ molecular subtypes would limit their use as a forecasting tool. Nevertheless, Tiriac and others have demonstrated that organoids represent an efficient drug-screening platform that could predict responses observed in patients to the common chemotherapy used in PDAC (Tiriac et al., 2018). Interestingly, while classical and basal-like signatures could be identified in the organoids, the authors described organoid-derived gene signatures that are unrelated to the transcriptional phenotypes and that could predict patient’s response to specific compound (Tiriac et al., 2018). Further major concern for clinical implementation is the absence of autologous stromal elements (endothelial cells, fibroblasts, immune cells) in most organoid culture systems. Even if this can be partially rescued by a reconstituted TME using patients’ derived cells, we still do not know whether culture conditions alter the stability and the phenotypes of stromal cells or even if clonal selection occurs in culture.

There are many ongoing efforts in the field, including our own (https://precode-project.eu/) trying to improve aspects of the organoid technology. For example, a recent report from the Jørgensen’ group (Below et al., 2021) described a fully-synthetic hydrogel that supported tumour organoid propagation and co-cultivation with stromal elements, thus promising to be transformative for the field. Moreover, modifying medium formulations can “push organoids back” to a phenotype that more accurately resembles their origins (Raghavan et al., 2021). These recent advances will likely accelerate organoids implementation in clinical practice and promote a wider adoption in the scientific community.

## Concluding Remarks

PDAC is an extremely deadly disease, whose biology, tumorigenesis, and progression we still do not fully understand as reflected by the limited therapy options and poor prognosis. PDAC might progress from a classical to basal-like/squamous phenotype through genetic or epigenetic dysregulations, influenced by intrinsic and extrinsic factors, that cause loss of pancreatic endodermal fate. However, the basal-like programmes might already exist within the normal pancreas and the disease progression might be dependent on pre-existing cell populations, which initiate the cancer. To understand lineage relationships and plasticity in this cancer and how they affect progression and therapy resistance, organoids and organotypic cultures have emerged as a valuable tool that holds promise to offer insights into PDAC, reveal novel targets, and bring tangible changes to patients’ management.
